# Contemporary issues on the epidemiology and antiretroviral adherence of HIV-infected adolescents in sub-Saharan Africa: a narrative review

**DOI:** 10.7448/IAS.18.1.20049

**Published:** 2015-09-16

**Authors:** Olurotimi A Adejumo, Kathleen M Malee, Patrick Ryscavage, Scott J Hunter, Babafemi O Taiwo

**Affiliations:** 1 Department of Child and Adolescent Psychiatry, University College Hospital, Ibadan, Nigeria; 2 Department of Psychiatry and Behavioral Sciences, Northwestern University Feinberg School of Medicine, Chicago, IL, USA; 3 Institute of Human Virology, University of Maryland School of Medicine, Baltimore, MD, USA; 4 Department of Psychiatry and Behavioral Neuroscience, University of Chicago, Chicago, IL, USA; 5 Division of Infectious Diseases, Northwestern University, Chicago, IL, USA

**Keywords:** adolescents, sub-Saharan Africa, HIV, adherence, antiretroviral, review, epidemiology

## Abstract

**Introduction:**

Adolescents are a unique and sometimes neglected group in the planning of healthcare services. This is the case in many parts of sub-Saharan Africa, where more than eight out of ten of the world's HIV-infected adolescents live. Although the last decade has seen a reduction in AIDS-related mortality worldwide, largely due to improved access to effective antiretroviral therapy (ART), AIDS remains a significant contributor to adolescent mortality in sub-Saharan Africa. Although inadequate access to ART in parts of the subcontinent may be implicated, research among youth with HIV elsewhere in the world suggests that suboptimal adherence to ART may play a significant role. In this article, we summarize the epidemiology of HIV among sub-Saharan African adolescents and review their adherence to ART, emphasizing the unique challenges and factors associated with adherence behaviour.

**Methods:**

We conducted a comprehensive search of online databases for articles, relevant abstracts, and conference reports from meetings held between 2010 and 2014. Our search terms included “adherence,” “compliance,” “antiretroviral use” and “antiretroviral adherence,” in combination with “adolescents,” “youth,” “HIV,” “Africa,” “interventions” and the MeSH term “Africa South of the Sahara.” Of 19,537 articles and abstracts identified, 215 met inclusion criteria, and 148 were reviewed.

**Discussion:**

Adolescents comprise a substantial portion of the population in many sub-Saharan African countries. They are at particular risk of HIV and may experience worse outcomes. Although demonstrated to have unique challenges, there is a dearth of comprehensive health services for adolescents, especially for those with HIV in sub-Saharan Africa. ART adherence is poorer among older adolescents than other age groups, and psychosocial, socio-economic, individual, and treatment-related factors influence adherence behaviour among adolescents in this region. With the exception of a few examples based on affective, cognitive, and behavioural strategies, most adherence interventions have been targeted at adults with HIV.

**Conclusions:**

Although higher levels of ART adherence have been reported in sub-Saharan Africa than in other well-resourced settings, adolescents in the region may have poorer adherence patterns. There is substantial need for interventions to improve adherence in this unique population.

## Introduction

Although significant progress has been achieved in understanding the pathogenic mechanisms, transmission, clinical features and complications of HIV/AIDS, the disease remains a global menace with large numbers of new infections and no scalable cure. Approximately 35 million people in the world currently live with HIV [1], over 7 million of these being children and youth aged less than 24 years [1].

Adolescence is a stage of life during which individuals have unique psychological, social and health needs. The defined age range of adolescence varies, but is generally accepted to begin with puberty and end in the transition to adulthood [2–4]. Rapid physical and hormonal development during adolescence is sometimes accompanied by a desire for self-discovery, an emerging sense of autonomy, separation from caregivers and the assertion of independence, as well as a quest for recognition and acceptance, which could lead to risk-taking behaviour [5,6].

Often portrayed as the “future generation” or the “next generation of adults” [3], adolescents are central to the evolution of social norms and values, and play important roles in shaping economic trends in society [7]. Adolescents also constitute a disproportionally large proportion of the population in countries with widespread poverty, political instability, rapid urban growth, civil strife, or natural disasters [3]. Adolescents in these settings frequently lack adequate social and economic support, as well as comprehensive health services.

Developmental characteristics of adolescents and societal conditions have implications for healthcare planning. The predisposition to impulsive behaviour and risks of mental health problems including depression and anxiety [8–10], as well as peer influence and risky behaviour [5,6] increase adolescents’ vulnerability to a variety of hazards, such as HIV/AIDS [8,11,12]. Other age-specific factors that may present a risk for HIV infection, or a challenge to its management, include immature judgment [13] and an evolution of the role of caregivers in HIV medication management and treatment. Caregivers may withdraw their support completely, show inconsistent support, or remain completely involved, with resulting implications for the adolescent's adherence to treatment [14–16]. Despite these common aspects of this developmental stage, adolescents are rarely considered a unique group in public health planning in sub-Saharan Africa, as their needs are typically overlooked or not fully recognized [3,17].

In this article, we review adherence to antiretroviral therapy (ART) among adolescents living with HIV in sub-Saharan Africa, their unique challenges, factors identified to affect their adherence behaviour and interventions evaluated to date. We provide recommendations for age-appropriate strategies to improve adherence in this population.

## Methods

We conducted a comprehensive query of Medline, PubMed and Google Scholar using search terms including “adherence,” “compliance,” “antiretroviral use” and “antiretroviral adherence,” in combination with “adolescents,” “youth,” “HIV,” “Africa,” “interventions” and the MeSH term “Africa South of the Sahara.” Also, relevant articles were retrieved from reference lists of the articles identified through this search method. In addition, relevant abstracts and reports from meetings held between 2010 and 2014 were queried and included if they related to work published before or during this period. In total 19,537 articles were retrieved, of which 215 were considered relevant and 148 were reviewed.

## Discussion

### HIV/AIDS among adolescents in sub-Saharan Africa

Approximately 2.1 million new HIV cases were reported worldwide in 2013 [1]. Children and young people aged 0 to 24 years are disproportionately affected, particularly in the sub-Saharan African region (Table 1) [18]. In 2012 and 2013, adolescents aged 10 to 19 years in sub-Saharan Africa were estimated to account for more than 80% of the world's entire population of adolescents living with HIV [19,20]. Sub-Saharan Africa also contributed significantly to the number of new adolescent infections worldwide in 2012, with 10 countries in this region making up nearly 70% of the new infections in 0 to 14 years olds that year [1].

**Table 1:** Number of people aged 0 to 14 years living with HIV and prevalence by sex among 20 to 24 year olds in 2013, by Joint United Nations Programme on HIV/AIDS

	Estimates of young people living with HIV, 2013			
	
Region	Children and adolescents 0 to 14 years (% of total no.) with HIV	Estimated number living with HIV (all ages)	Percent of 20 to 24 year-olds with HIV (females)	Percent of 20 to 24 year-olds with HIV (males)
Sub-Saharan Africa	2,900,000 (11.7)	24,700,000	2.2	1.1
Asia and the Pacific	210,000 (4.4)	4,800,000	<0.1	<0.1
Caribbean	17,000 (6.8)	250,000	0.5	0.4
Eastern Europe and Central Asia	14,000 (1.3)	1,100,000	0.2	0.2
Latin America	35,000 (2.2)	1,600,000	0.1	0.3
Middle East and North Africa	16,000 (7.0)	230,000	<0.1	<0.1
Western and Central Europe and North America	2800 (0.1)	2,300,000	<0.1	0.2
GLOBAL	3,200,000 (9.1)	35,000,000	0.4	0.3

Joint United Nations Programme on HIV/AIDS (UNAIDS), Epidemic Monitoring and Analysis, Gap Report and 2013 estimates.

**Figure 1:**
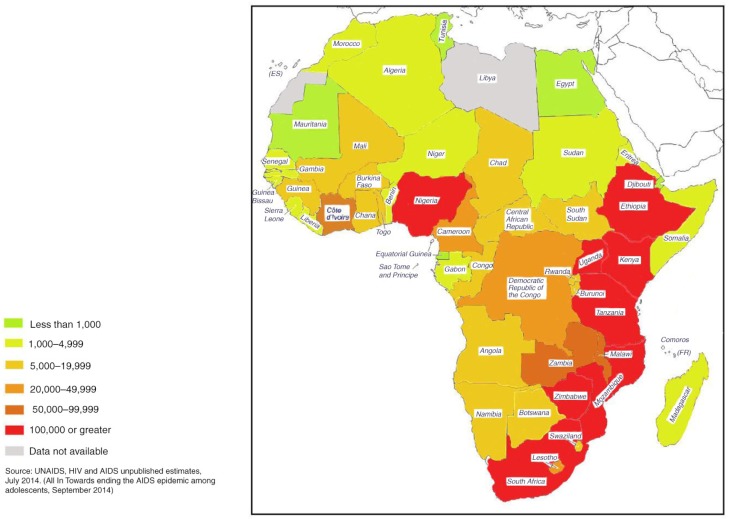
Map of Africa showing estimated number of adolescents aged 10 to 19 years living with HIV in Africa by country in 2013.

The distribution of HIV/AIDS among adolescents across sub-Saharan Africa is uneven (Figure 1). In 2009, an estimated one in every three young people newly infected with HIV was from South Africa or Nigeria [21], two of the countries with the world's largest HIV-infected populations. About 1.3 million adolescents currently live with HIV in East and Southern Africa, whereas 390,000 live in West and Central Africa 19. Because children and youth constitute large proportions of the population in many sub-Saharan African countries [22,23], HIV/AIDS among these young populations is a proximate threat to societies and economies in the subcontinent.

#### HIV and stigma

Individuals living with HIV/AIDS have been the subject of stigmatizing attitudes and differential treatment in nearly every part of the world, including the sub-Saharan African region. For several years, widespread misconceptions about the disease contributed to its portrayal as a result of divine punishment [24,25], witchcraft [26], or an invariable outcome of promiscuous sexual behaviour [25,27,28], which always resulted in death. These perceptions are believed to have contributed to the persistently high rates of spread in the sub-Saharan African region compared to elsewhere, primarily through individuals’ and families’ efforts to avoid being identified with HIV. As such, individuals with HIV avoid voluntary testing, and women insist on breastfeeding their babies to avoid suspicion in several African societies [29–32].

With increased availability of life-saving antiretroviral treatment, consequent improved HIV survival across age groups and widespread stigma reduction campaigns [33–35], HIV/AIDS has come to be viewed in a less negative light in several African communities. Nevertheless, recent studies among adolescent populations in this region reveal persisting high rates of stigma. Wolf [36], for instance, identified discriminating attitudes against Kenyan youths with HIV from their peers and school teachers. Such stigmatizing behaviour as name-calling and avoidance are still associated with loss to follow-up, poor adherence, failure to disclose status, decisions to drop out of school, avoidance of antenatal care and testing by pregnant female adolescents, and even depression and suicidal ideation among sub-Saharan African youth with HIV [36–39]. These potential effects highlight a need for further research to understand the ways in which stigma interacts with treatment behaviour among youth, with a view to developing services to address these among youth in this region.

### Sex differences in HIV/AIDS among sub-Saharan African adolescents

The highest HIV prevalence rates among youth in the United States and parts of Europe are found among male gay and bisexual adolescents and young adults, particularly those with African ancestry [18,40]. In contrast, higher rates are reported among female than male youth in sub-Saharan Africa [12]. According to UNICEF reports [41], females aged 15 to 17 years in this region have up to four times the prevalence rates of HIV reported among their male counterparts. Female adolescents may be infected by male partners through “intergenerational sex” occurring in contexts of power imbalances, poverty, manipulation or exploitation, and without condom use [42,43]. Men in such relationships often have multiple sexual partners and acquire sexually transmitted infections, which they pass on to adolescent females [44]. Lesotho and Swaziland in southern Africa provide a striking picture of these realities. Reports indicate an average HIV prevalence of 6.0% in female adolescents aged 15 to 17 years in Lesotho and in Swaziland. These rates increase to 30% in Lesotho and more than 40% in Swaziland among young women aged 23 to 24 years [43]. These high female rates may also be related to unprotected heterosexual relations with multiple partners, sometimes in concurrent relationships [44].

An additional factor contributing to the sex imbalance in adolescent HIV prevalence may be the two-fold or higher risk of AIDS-related mortality in male adolescents compared to females, which may be related to the lower proportion of males who receive ART [45,46].

Young gay men and other young men who have sex with men (MSMs) are a significant high risk group for HIV/AIDS. Although Global AIDS Response Progress Reporting data from 96 countries indicate a median HIV prevalence of 3.7% among MSMs of all ages, the prevalence is about 4.2% in those aged below 25 years [47]. Recent global AIDS reports reveal higher median HIV prevalence rates among MSMs in west and central Africa (15%), and in eastern and southern Africa (14%), compared to other regions of the world (6 to 13%) [47]. However, precise data on HIV prevalence trends among this population are unavailable in many parts of Africa [1], likely related to stigma and in some cases criminalization of same-sex relationships.

### Perinatally- versus horizontally-infected adolescents

In 2007, it was estimated that up to 90% of all children with HIV aged under 15 years had become infected through their mothers during pregnancy, labour, delivery, or via breastfeeding [48]. There have been significant reductions in perinatal transmission of HIV in both well-resourced and resource-limited settings in recent years [1,49] and improved treatment with combination ART has resulted in large numbers of children surviving into adolescence and beyond [50–53]. However, perinatally-infected children who reach adolescence may have experienced chronic immunosuppression, which has been associated with impaired neurocognitive development and delayed sexual maturation [54,55]. They are also at risk of long-term ART adverse effects, including hyperlipidaemia, cardiovascular disease and renal impairment [50,56,57], and may experience reduced efficacy of combined oral contraceptive pills due to interactions with ARTs [58].

In addition to the large population of perinatally-infected adolescents, a substantial number acquire HIV through other routes such as sex and injecting drug use. Among young people aged 10 to 24 years all over the world, most HIV infections are believed to be sexually acquired [59]. HIV-infected adolescents in several parts of sub-Saharan Africa are unaware of their HIV status [21,49], and lack access to counselling, testing and treatment needed to prevent onward transmission [49,60]. Even where such facilities are available, adolescents may fall below the legal age of independent consent for these services [61].

### Mortality and morbidity among HIV-infected youth in sub-Saharan Africa

In contrast to the significant decline in global deaths from AIDS-related causes over the past decade [1,18,49], deaths among adolescents have increased during this period [62,63]. HIV currently ranks second among global causes of adolescent deaths [64], and one study found a nearly 50% increase in adolescent AIDS-related deaths between 2005 and 2012 [12]. This increase has occurred predominantly in the African region.

Neurocognitive deficits and psychiatric symptoms are complications among individuals of all ages with HIV and have implications for adherence. Several studies conducted in well-resourced settings have reported a high prevalence of neurocognitive and psychiatric morbidity among HIV-infected adolescents compared to those uninfected [65–67]. In addition, emotional and behavioural problems are more frequently observed among HIV-infected adolescents, compared to normative data or comparison groups [68]. Rates, however, vary across studies, with some researchers reporting no difference between infected and uninfected groups, or even more psychological problems among uninfected adolescents than those infected [68]. Most research into neuropsychological outcomes of HIV/AIDS in sub-Saharan Africa has focused on adult [69–74] or paediatric [75–78] populations, and there is a need for adolescent studies in this area.

### Adherence to ART

Adherence to ART in HIV-infected individuals is a strong determinant of disease outcome. Interventions which improve adherence are associated with successful viral suppression, reduced risk of opportunistic infections and prevention of drug resistance [79–81]. Although adherence levels as low as 80% have been associated with treatment success, adherence of around 95% is widely considered desirable for viral suppression and prevention of ART resistance [82,83]. Studies all over the world, including in sub-Saharan Africa, have identified adolescents with HIV as being at particular risk of poor adherence [84–86].

### Measures of adherence

A gold standard measure of ART adherence remains elusive. Some assessment methods are easily applied because they are a part of routine clinical care, but some research studies have chosen certain methods based on perceived benefits over others. In sub-Saharan Africa, ART adherence studies among youth have used both direct and indirect assessment methods [87,88]. Table 2 summarizes the strengths and weaknesses of some of these measures.

**Table 2:** Some measures of antiretroviral adherence used in sub-Saharan African studies, with merits and drawbacks

Adherence measure	Strengths	Drawbacks	Comments
*“Direct” measures*			
Plasma drug assays	Accurate and relatively objective	Limited laboratory resources in several low-resource settings	Used in relatively few studies in SSA
	Demonstrated to correlate with immunologic response in Tanzanian children and adolescents [111]	May only give information about a given time-point, and not long-term adherence	Pharmacokinetic factors may cause inter- and intra-patient variations in drug assays [112]
		Reliability subject to host pharmacokinetic factors	
		Relatively high cost	
Directly observed therapy	Actual ingestion of ART can be monitored	No demonstrated efficacy over self-administered ART in a study of South African adults [113]	Mainstay of tuberculosis treatment recommended for use in adolescents on ART [114–116]
	Successfully adopted to improve ART adherence in Kenya [117,118]	May be time consuming in busy clinic settings	
*“Indirect” measures*			
Self-report	Easy to obtain during routine clinic visits	Adherence prone to inadvertent or deliberate overestimation by patients [89,111]	Most widely used adherence measure in SSA [111,119–121]
	Relatively inexpensive	Social desirability and recall bias may contribute to inaccuracy	
	Easily supported by aids like visual analogue scales		
	Demonstrated to correlate with virologic outcomes in Uganda		
Electronic monitoring methods and devices	Some forms (MEMS) demonstrated to correlate with virologic suppression in Uganda and South Africa [125,126]	Expensive [122–124]	Electronic-operated pill-containing devices record and/or transmit data each time an ART dose is taken out. Most common devices use microchips incorporated into pill bottle caps
*Pharmacy-based measures*			
Pill count	Practical, easy to obtain at clinic visits	Easily manipulated; dependent on patient's cooperation [127]	Patients return unused pills at each pharmacy visit, and count of unused pills indicates doses missed after last drug refill
	Demonstrated to be a valid adherence measure among adolescents in Botswana [129]	Time-consuming and inconvenient in busy clinic settings [128]	
	Unannounced home-based counts possible, and may improve reliability [127,130]	Patients may forget to turn in unused pills	
Pharmacy visits/medication refills	Easy to obtain	May not accurately reflect ART use, for example, in patients who dump pills or accumulate them without using	Medications are dispensed to cover the exact period between visits, and delayed return dates are taken to be indicative of missed doses
	Inexpensive	Patients’ use of multiple pharmacy sources may make measure unreliable	Use of pharmacy refill data useful for computing MPR, a valid adherence measure in low-resource regions [131–133]
	Useful in low-resource settings [134]		

“Direct” measures, methods which provide objective evidence of patients having ingested medication [87,88]. “Indirect” measures, methods which infer frequency of medication use based on an observable indicator [87,88]. SSA=sub-Saharan Africa; ART=antiretroviral therapy; MEMS=Medication Event Monitoring System; MPR=Medication Possession Ratio.

In a study among Ugandan adolescents with HIV, pill count and self-report measures yielded significantly higher adherence values and were considered less accurate than electronic measurement methods [89]. On this basis, the investigators recommended the use of electronic methods as a possible gold standard for measuring ART adherence in research [89]. Other researchers recommend that multiple methods such as combinations of caregiver and youth self-reports, pill count and pharmacy records be used to assess antiretroviral adherence, especially in young populations [90].

### Patterns of adherence in sub-Saharan African adolescents with HIV

Antiretroviral adherence patterns in adolescents vary across different regions of the world. In a recent systematic review and meta-analysis, Kim *et al*. [91] found that adolescent adherence was poorer in North America and Europe than in less-developed settings like Africa and Asia (see studies in Table 3). In spite of this, poor adherence behaviour among adolescents in sub-Saharan Africa is a significant concern, given the limited ART options available in most parts of the subcontinent, and the risk of drug resistance [92].

**Table 3:** Summary of reported rates of antiretroviral adherence among children and adolescents in sub-Saharan African countries and other regions

Study	Location	Adherence measure	Findings
*Studies in West Africa*			
Elise *et al*. [168]	Cote d'Ivoire	Clinic attendance, self or caregiver report	67% of children aged 13 to 17 years had missed no doses in the previous month
Iroha *et al*. [169]	Nigeria	Caregiver report	86.3% of a sample of children and adolescents had been 100% adherent in the previous 3 days
Mukhtar-Yola *et al*. [170]	Nigeria	Caregiver report	80% of children aged 1 to 15 years had ≥95% adherence; 62.5% reported 100% adherence
Polisset *et al*. [171]	Togo	Caregiver report	Among sample aged 1 to 14 years, 14% ≥10 years 42% had no missed doses over previous 4 days or previous month
Ugwu and Eneh [172]	Nigeria	Self-report	76.1% of children and adolescents aged 5 months to 17 years had >95% adherence; 59.2% reported 100% adherence
*Studies in East Africa*			
Biadgilign *et al*. [139]	Ethiopia	Caregiver report	Patients aged 3 to 14 years with adherence ≥95%: Same day, 98.2% Day before, 96.9% Previous 3 days, 93.1% Previous 7 days, 86.9%
Biressaw *et al*. [121]	Ethiopia	Caregiver report (CR);	Children aged 8 to 13 years: CR: 90% reported 100% in past month
		Unannounced pill count (uPC)	93.3% had ≥95% adherence in past 7 days uPC: 34.8% had ≥95% in past 7 days
Byakika-Tusiime *et al*. [173]	Uganda	Three-day caregiver-report (SR) 30-day visual analogue scale (VA)	Mean adherence for children initiating ART (I) and children on long-term treatment (L) SR: I, 98.1%; L, 100%
		Unannounced pill count (uPC)	VA: I, 97.8%; L, 100%
			PC: I, 100%; L, 87.7% All patients had ≥95% adherence by SR, but only 36% had ≥95% by PC
Langat *et al*. [90]	Kenya	Pill/drug count (PC)	Patients 3 to 14 years; average adherence 44.2% PC: 27% had 100% adherence
		Caregiver report (CR)	CR: appointments kept 45.7%
		Drug refill data (DR)	Appropriate timing of doses 56.1% DR: 47.8% overall adherence
Mghamba *et al*. [111]	Tanzania	Caregiver report (CR)	Children 2 to 14 years CR: 98% missed <1 dose in past 3 days
		Pill count (PC)	PC: 97% returned <5% previous dispensed pills
		Nevirapine plasma assay	85% had nevirapine concentration >3 µg/ml
Nabukeera-Barungi *et al*. [120]	Uganda	Three-day self-report (SR)	Children and adolescents 2 to 18 years SR: 89.4% of sample had ≥95% adherence
		Pill count (PC)	PC: 94.1% had ≥95% adherence
		Unannounced pill count (uPC)	uPC: 72% had ≥95% adherence
Ndiaye *et al*. [129]	Botswana	Pill count	Adolescents 13 to 18 years Overall median adherence 99% 76% had >95% adherence
Wamalwa *et al*. [174]	Kenya	Caregiver report (over past 3 days or 2 weeks)	Among children 8 months to 12 years: 64% had 100% adherence
Vreeman *et al*. [142]	Kenya	Self/caregiver report	71% of children aged 1 to 14 years missed at least one dose over a 3^3/4^ years observation period. Odds of non-adherence higher with death of both parents
Wiens *et al*. [89]	Uganda	Self-report (SR) Pill count (PC)	Adolescents 12 to 17 years: SR: 99% adherence overall; 93% had >95%
		eCAP™	PC: 97% adherence overall; 67% had >95%
			eCAP^TM^: 88% overall; 23% >95% adherence
*Studies in Southern Africa*			
Nachega *et al*. [84]	South Africa	Pharmacy refill at 6, 12, and 24 months	Adolescents 10 to 19 years vs. adults 20 and above 6 months: 20.7% (vs. 40.5% in adults) 12 months: 14.3% (vs. 27.9% in adults) 24 months: 6.6% (vs. 20.6% in adults) [*p*<0.01]
Reddi *et al*. [175]	South Africa	Child and caregiver report	Children 4 months to 15 years
			89% of patients reported >95% adherence
			59.6% had 100% adherence
aSummary estimates from studies in other regions			
*North America*			
22 studies		Viral load, self-report, MEMS	62.3% overall adherence (95% CI 57.1 to 67.6)
Asia			
*3 studies*		Viral load, self-report	83.9% overall adherence (95% CI 76.8 to 91.0)
Europe			
12		Viral load, pill count	62.0% overall adherence (95% CI 50.7 to 73.3)
*South America*			
5		Viral load, self-report	62.8% overall adherence (95% CI 46.6 to 77.0)

a From Kim *et al*. [91]. CI=confidence interval; MEMS=Medication Event Monitoring System; eCAP™=electronic medication vials.

Compared to other age groups with HIV, adolescents in developed settings are reported to have poorer ART adherence [93–96], a pattern similar to that reported between adolescents and adults in sub-Saharan Africa [84,97–99]. There is also some evidence that adolescents aged 15 years and older are at higher risk of poor adherence than children and younger adolescents in sub-Saharan Africa [100], as has been described elsewhere [101]. The transfer of responsibility for treatment from caregivers to adolescents themselves is likely implicated.

Most studies on adolescent ART adherence from sub-Saharan Africa either fail to distinguish between perinatally- and horizontally-infected patients, or focus exclusively on perinatally-infected groups. Findings from some studies in developed settings suggest that horizontally-infected adolescents may have poorer ART adherence than those infected perinatally [102,103].

### Factors influencing antiretroviral adherence among adolescents

A considerable amount of the literature on antiretroviral adherence in sub-Saharan Africa has focused on factors which influence adherence behaviour. Numerous factors have been identified, several of which are believed to act simultaneously. The weight of influence of various factors also varies based on socio-economic, cultural and environmental characteristics in different settings.

#### Socio-demographic factors and individual resilience factors

Associations have been found between socio-demographic factors such as age and living conditions, and adherence in some African settings. In a study of 314 Ethiopian youth receiving care in tertiary ART facilities, adherence levels were significantly poorer among older children and adolescents, compared to younger children [102]. Nachega *et al*. [84] also reported lower rates of adherence among adolescents compared to adults in a comparison study of almost 8000 patients receiving ART in South Africa. Mutwa *et al*. [104] highlighted the impact of living situations on adherence to ART among 42 perinatally-infected adolescents in Rwanda. Adolescents who lived in boarding houses, foster care, or orphanages were often faced with a lack of privacy, lack of support, or stigma if they were discovered to be using ARTs, which made it difficult to maintain medication use.

Several studies identify individual adherence-enabling factors among adolescents in sub-Saharan Africa. High levels of ART adherence were reported among South African youth, who attributed their ability to cope with their HIV-positive status to the availability of ARTs and the ability to maintain positive attitudes [105]. Individual competence, arising from high levels of cognitive functioning and good adaptive skills, also appears to help individuals cope with HIV-related stressors [106]. Furthermore, among youth living with HIV, good psychological adjustment [107,108] and positive future expectations play protective roles [109,110]. Although little is known about the influence of these resilience factors on ART use patterns, they may act to improve adherence among adolescents.

#### Structural and economic factors

Structural or economic factors in sub-Saharan countries may pose barriers to adherence. Jimmy-Gama *et al*. [135,136] reported that challenges to youth uptake of ARTs in Malawi included the unavailability of food, a factor also identified among adolescents in the Democratic Republic of Congo. Similarly, lack of nutritional support was identified as a reason for poor ART adherence among adolescents in urban Ethiopian settings [137]. In the Malawi study, regular access to ART was also difficult due to unaffordable but compulsory treatment fees [135]. In a study among 440 young adolescents receiving ART at district hospitals in northeast Ethiopia, living in close proximity to a treatment centre was associated with better ART adherence [138]. Other researchers described difficulties with access and cost of transportation as economic barriers to adherence among adolescents in an urban Ethiopian setting [139]. These economic challenges sometimes arise from families’ loss of livelihood following the death of members from HIV/AIDS [140] and represent some of the multiple structural barriers to ART adherence in socio-economically deprived parts of sub-Saharan Africa. The loss of a family member may also pose an obstacle to adherence for perinatally-infected adolescents who invariably depend on caregivers for treatment [141,142] and may have to abruptly assume responsibility under such circumstances.

The negative impact of civil disruptions due to political instability and violence on healthcare provision has been well documented by several researchers [143–146]. Several communities in sub-Saharan Africa have experienced significant political violence in recent decades, including the 1986 political conflicts in South Africa [147,148], and more recently, the Kenyan post-election conflicts [144,149,150]. Such situations of violence have negatively influenced health through consequences of death, disabilities, displacement and destruction of health facilities and supplies [145]. Specifically, political violence has been associated with significant disruptions in HIV patient care in Africa through widespread fear, lack of transportation, physical attacks and displacement of individuals with HIV within affected communities [144,149–151]. In communities at risk of political violence, it is helpful for HIV treatment programmes to have contingency measures, such as emergency preparedness plans in conjunction with local agencies, to forestall treatment interruptions in the event of such outbreaks [151].

#### Psychosocial factors

The importance of social support to ART adherence has been highlighted in many sub-Saharan African studies. Fetzer *et al*. [136] described a strong association between caregiver supervision and ART adherence among adolescents in the Democratic Republic of Congo. Similar associations have been reported in qualitative studies among young adolescents with HIV infection and their caregivers in Kenya, Uganda and South Africa [152–154]. Among a sample of South African adolescents, those with extensive supportive networks among relatives and peers appeared to cope better with psychosocial challenges, and caregivers played an important role in facilitating ART adherence. The participants in this study opined that caregivers contributed to their good adherence by reminding them to take their medications [105].

Where caregiver involvement declines [155], the ability of hitherto dependent perinatally-infected adolescents to assume responsibility for their treatment may be threatened by developmental, psychological and social factors. Assuming responsibility for HIV treatment unlike their age mates may conflict with adolescents’ desire for peer acceptance and approval, which can be compounded by stigma, socio-economic challenges and treatment fatigue [136,137,152].

Among horizontally-infected individuals, poor adherence has also been associated with complicated medication routines, as well as individual factors such as forgetfulness and mental health problems [85,103,156]. Psychosocial problems including non-recognition of a need for medications, fear of disclosure, poor social support and involvement in risky behaviour such as substance use, have also been identified among adolescents with behaviourally acquired HIV in the United States [157–159].

##### Disclosure

Disclosure of HIV status has been studied in different contexts, two of which have been repeatedly associated with adherence behaviour. As adolescents mature, their evolving social relationships may require them to provide details about their HIV-infected status to their peers or intimate partners (self-disclosure). This is often a challenge, especially in settings where HIV/AIDS remains stigmatized [160]. Mutwa *et al*. [104] noted that adolescents’ fear of discovery and reluctance to disclose their status made them avoid taking their ARTs in non-private settings like boarding houses and foster homes.

Caregiver disclosure of adolescents’ HIV infection status is a more frequently studied form of disclosure [161,162] and is also challenging, as evidenced by findings that only 38% of adolescents aged 11 to 15 years in a Zambian study had been informed about their HIV status [163]. Similarly, Bikaako-Kajura *et al*. [153] found that among 42 Ugandan youth aged 5 to 17 years, only 29% had had their HIV status fully disclosed to them by their caregivers, and only 38% had received partial information about the reason for their frequent illnesses and need for repeated medication. In both of these studies, ART adherence was poorer in children who had not been disclosed to, especially in older adolescents. Bikaako-Kajura *et al*. [153] found that adherence in such situations was often completely dependent on the caregiver and speculated that these adolescents wilfully missed doses whenever possible, as if in rebellion against their caregivers’ secrecy or lack of full disclosure. Similarly, in a study among Ugandan children aged 2 to 18 years, Nabukeera-Barungi reported that children or adolescents were three times more likely to be non-adherent when their caregiver was the only one who knew their HIV status [120]. Several other researchers document associations between early disclosure and satisfactory adherence patterns. In a qualitative study among adolescents aged 10 to 19 years in Zambia, Mburu *et al*. [164] reported that among other effects, caregivers’ disclosure created opportunities for improved adherence support for the adolescent. Fetzer *et al*. [136] described reduced levels of frustration among adolescents that had been disclosed to, because disclosure provided a motivational factor aiding adherence.

##### Stigma

Among caregivers who hesitate or avoid disclosing children's HIV status, the fear of exposing the child or adolescent to stigma is often cited as a reason [164,165]. Socio-cultural misperceptions about the aetiology and spread of HIV/AIDS accentuate the effect of stigma in some parts of sub-Saharan Africa [29,166]. In a qualitative study among caregivers and healthcare providers in Ethiopia, stigma within families was cited as a reason for ART non-adherence [137]. Children were sometimes not given medications at home to avoid stigma from relatives or neighbours who might be present at the time. In the Democratic Republic of Congo, shame and stigma were most frequently cited as barriers to adherence by adolescents who recognized that taking ART made them different from others and exposed them to ridicule [136].

Adolescents are often concerned about “feeling normal” and not feeling “different from their peers.” Apart from the inherent difficulty of repeatedly taking medications, adolescents sometimes skip ART doses because they are a reminder of a condition that makes them different from others [136]. Thus, ART adherence can be a paradoxical source of stigma, as is supported by the findings of Makoae *et al*. [167] in five African countries. The researchers compared groups of HIV-infected individuals taking ART medications, with groups without medications in five countries – Lesotho, Malawi, Swaziland, Tanzania and South Africa over five time points. Measuring levels of HIV-related stigma at six-month intervals on the HIV/AIDS Stigma Instrument-PLWA (HASI-P), they observed an increase in perceived stigma among individuals taking ART, compared to those not taking [167].

In contrast to this report, studies in Kenya and Uganda [117,176] have demonstrated decline in internalized stigma among adult patients after a period on ART. This is supported by the findings of qualitative studies among people with HIV in Zimbabwe and South Africa, who generally attributed their improved self-image, functioning and wellbeing to the role of antiretroviral treatment [177–179]. Improvements in physical and mental health were associated with reduced internalized stigma in the Ugandan study, suggesting that the effect of ART adherence on reducing stigma may be mediated through improvements in quality of life in these African populations [176]. It is plausible that optimal ART adherence may influence stigma reduction among adolescents similarly if adolescents experience improved health and wellbeing with antiretroviral medication use.

#### Individual factors

Several studies have reported “forgetting to take medications” as a reason for skipped doses, especially in situations when the adolescent is free from acute illness. For most HIV-uninfected adolescents, day-to-day living does not include medication use, and the absence of memory aids can result in forgotten doses for those youth with HIV receiving ART. Among a sample of older adolescents and adults attending an outpatient clinic in the Democratic Republic of Congo, responses to a standardized questionnaire indicated challenges to ART adherence [180]. These included forgetfulness and difficulty in organizing a schedule around medication use [180], factors which may also be related to subtle deficits or impairment in memory, cognitive and executive function, or behavioural-emotional difficulties that often occur in the background of HIV/AIDS [68,181–184]. Some research, including studies on offspring of HIV-infected mothers in Africa, has highlighted the risk of neurocognitive delay in infants and children infected or affected by HIV [76,185–187]. These deficits may be indicative of early neurotoxic effects of HIV on the developing central nervous system of individuals exposed to HIV *in utero* or during early childhood, resulting in lasting deficits that may also compromise adherence during adolescence [188]. However, other studies point to subtle challenges in early language development being the possible effect of *in utero* exposure to ART use in pregnancy [189,190]. Furthermore, adolescents with HIV infection are frequently exposed to adverse environmental influences including poverty, stress, violence and maternal ill-health, which could also contribute to neurocognitive and psychiatric risk [191,192]. These findings suggest a multifactorial aetiology to neurocognitive and behavioural outcomes among this vulnerable population, and there is the need for further research to ascertain the contributions of these individual risk factors.

#### Treatment-related factors

Treatment-related factors, including real or anticipated side effects and having to take large quantities of drugs (“pill burden”), have been cited as barriers to ART adherence among children and young adolescents [137,172,180]. Pill burden has been reported to hinder ART adherence among youth populations in the United States [156,193]. Pill burden was also mentioned as the most common reason for skipping ART doses among a sample of adolescents in South Africa [84]; increased burden from the medications prescribed for coexisting conditions contributes further to poor adherence.

A potentially valuable intervention for these medication-related challenges among adolescents is the use of long-acting antiretroviral agents. Over the past few years, a number of new formulations, notably rilpivirine (a non-nucleoside reverse transcriptase inhibitor) and GSK1265744 (an HIV integrase inhibitor), have been developed for potential use at nearly 30-day intervals [194–196]. Surveys have demonstrated widespread acceptability of these agents among adults with HIV due to the potentials for ease of dosing. These agents are currently still in developmental stages, and although yet to be approved for regular use, represent a significant breakthrough particularly for poorly adherent populations of persons with HIV, among whom adolescents constitute a significant subgroup. Initial access to these new agents may be hampered by their considerable cost, particularly for adolescents in resource-poor settings like most parts of sub-Saharan Africa. Nevertheless, significant efficacy among adults has been reported for low doses of rilpivirine at low cost, making it a promising long-acting agent for use in resource-limited settings [194]; evaluation of efficacy among adolescents requires demonstration in future studies.

Transition of care between paediatric and adult HIV treatment services is sometimes challenging for adolescents with HIV in well-resourced settings. Youth with perinatally acquired HIV develop strong relationships with their paediatric care providers through their childhood and adolescent years, and are often reluctant to break these links in exchange for new, unfamiliar providers [197–200]. Transition to adult services is also challenging for developmentally unprepared adolescents, who may be emotionally or cognitively delayed as a complication of HIV infection [201], and who may be unable to access psychosocial support appropriate to their unique needs in adult care settings, compared to paediatric settings [200,202,203]. As a result, transiting adolescents are at risk of discontinuation of, or irregular access to ART. HIV care services in sub-Saharan Africa are distributed between specialized paediatric and adult clinics in some communities, but in other cases, general primary or secondary care facilities provide services for all age groups. Although anecdotal reports indicate that similar challenges exist in sub-Saharan Africa where youth are often transited from paediatric to adult HIV treatment programmes in mid-adolescence, there is little data on the challenges of transition and its impact on ART adherence in this region. Apart from the need to understand the experience of adolescents transiting in Africa, future research needs to focus on the experience of horizontally-infected youth, whose adherence challenges may differ from widely studied adolescents with perinatally acquired HIV [198,199].

### Consequences of poor adherence

Poor adherence to ART is associated with less effective viral suppression and reduced chance of survival in adolescents and other people with HIV [55,204,205]. In the study by Nachega *et al*. [84] comparing clinical outcomes of adherence among adolescents and adults in southern Africa, adolescents had poorer outcomes. Significantly fewer adolescents achieved complete adherence at each of three time points, and adolescents had lower rates of virologic suppression and immunologic recovery than adults [84]. Increased risk of morbidity and mortality arise from a host of complications of immune suppression and chronic HIV infection, such as opportunistic infections, cardiomyopathy and malignancies [206]. Other outcomes of poor adherence include the development of drug resistance and the risk of transmitting resistant strains of HIV to others when adolescents become sexually active [207,208]. Among effects associated with suboptimal ART adherence, some studies have reported impairments in neurocognitive functioning among adults with HIV [209,210], although there is a specific dearth of research into these associations among youth in sub-Saharan Africa.

### Interventions to improve adherence

Given the fact that barriers to adherence vary among societies, the success of adherence improvement interventions may depend on how well they are adapted to the unique challenges in each society. Similarly, adolescents constitute a unique, at-risk group whose interests and challenges may differ from those of other age groups, and likely require tailored interventions to improve adherence behaviour. In the sub-Saharan African region, few programmes for improving ART adherence exist for adolescents, and there is a dearth of research into the efficacy of interventions for this age group. The following subsection will, therefore, focus on the few existing interventions, most of which have been developed for adult populations.

A variety of strategies have been developed to improve adherence to ART in both well-resourced and low-resource settings. Although some of these strategies are based on cognitive or behavioural principles, others have involved direct observation and a number of interventions have involved “affective” strategies [211] (Table 4). In some settings, successful strategies introduced to promote retention in treatment have resulted in improved uptake of services [212], with resulting improved adherence across patient age groups. Strategies documented to be most effective in better-resourced settings are mostly patient-based, behavioural interventions [213,214] targeted at those identified to be poorly adherent [215]. A review of randomized controlled trials (RCTs) conducted between 1996 and 2005 also found that interventions associated with improved adherence outcomes were those which addressed practical medication management skills in the individual patient, and which were implemented over an at least 12-week period [214].

**Table 4:** Studies describing effective intervention programmes to improve ART adherence, specifically among adolescents with HIV in sub-Saharan Africa

Study	Location	Description of intervention	Category of intervention	Target
*East and Central Africa*				
Musiime *et al*. [243]	Uganda	Peer support group of adolescents living with HIV. Group held monthly meetings, had talks and discussions on a variety of health and treatment topics. Also, recreational activities leading to formation of a band aimed at reducing stigma and improving self-confidence. Counselling provided for adolescents with identified needs.	Affective	Adolescents
Van Winghem *et al*. [216]	Kenya	*“Axis 1”* Adopting a family-centred care approach in clinics; Tracing patients who missed appointments; Designated days for recreational activities; Age-relevant support groups for patients and caregivers; Adherence aids: pill boxes and tick sheets	Cognitive, behavioural, and affective	Adolescents, caregivers, and clinic staff
		*“Axis 2”* Development and use of a pocket-size booklet with educational information about HIV/AIDS; Individual counselling services		
		*“Axis 3”* Treatment literacy training Clinic staff training and support Incorporation of patients into clinic-based activities		
Ssewamala *et al*. [239]	Uganda	“SUUBI+Adherence,” a youth-focused economic approach to HIV treatment. Designed to improve ART adherence among youth in and out of school with HIV, through economic empowerment initiatives.	Economic	Adolescents
*Southern Africa*				
Bhana *et al*. [244]	South Africa	VUKA family-based programme: Educational material presented on psychosocial and treatment aspects of HIV/AIDS to adolescents and their caregivers in 10 sessions over 3 months.	Cognitive, affective, and behavioural prevention programme	Adolescents and caregivers
Fatti *et al*. [245]	South Africa	Lay community-based adherence support (patient advocates) conducted home visits to address household challenges affecting adherence over a 4-year period.	Affective	Caregivers
Mavhu *et al*. [246]	Zimbabwe	Three-component adolescent and family-centred programme: 1) Individualized support from community adolescent treatment supporters 2) Individual cognitive-behavioural therapy 3) Caregiver training to enhance adolescent support	Cognitive-behavioural, affective	Adolescents, caregivers

#### Interventions based on cognitive, behavioural and affective principles

In sub-Saharan Africa, some interventions based on cognitive and behavioural theories have been shown to be effective. One of the few existing adolescent-focused programmes was presented in a Kenyan study which described a three-pronged intervention targeted at adolescents, their caregivers, and care providers. This programme incorporated behavioural, cognitive and psychosocial strategies; a combination of interventions which appeared to foster adherence among adolescent participants [216]. In relation to these, education-focused strategies targeted at improving health literacy concerning HIV and ART use have been found to potentially improve adherence behaviour among youth in African countries including Ghana, Botswana and South Africa [217–220], similar to findings among similar populations in well-resourced settings [221–223].

Research findings among adolescents with HIV have also suggested that adolescents’ direct involvement in their own HIV treatment decisions may improve adherence behaviour [224–226]. In several sub-Saharan African societies, cultural influences on patient–physician relationships result in predominantly paternalistic-style relationships, which encourage patients to rely completely on their physicians for treatment-related planning and decision-making. There is little evidence to suggest that these relationships differ for youth with HIV in these cultures, who may be less likely to seek to participate in their own treatment decisions for fear of disrespecting their usually older care providers. There has, however, been limited research into the potential impact of these perceptions and practices on ART adherence.

#### Behavioural interventions

Purely behavioural interventions attempt to modify behaviour by reinforcing positive adherence patterns, through strategies such as memory aids. The use of reminder mobile-phone text messages uses such strategies, and its effectiveness has been demonstrated in RCTs among adult patients in Kenya [227,228]. The most common forms of observational monitoring evaluated for improving ART adherence involve direct observed treatment (DOT). Multiple studies in Kenya [229], Mozambique [230], South Africa [231] and Nigeria [232,233] demonstrated improved clinical outcomes in adult patients whose medication use was witnessed regularly by designated healthcare personnel or a family member.

#### Interventions involving affective approaches

Interventions classified as “affective” aim at improving ART adherence through emotional support [211,234] and include the use of psychotherapy or antidepressant medication. In Rakai, Uganda, peer health workers, themselves people living with HIV, were trained and equipped to conduct biweekly visits to assigned patients, with resulting improvements in adherence as reported by clinic staff [235]. Similar results were obtained using trained peers and “treatment partners” in Mozambique [236] and Nigeria [233]. Improvements in adherence have also been reported with the use of support groups, positive-living workshops and buddy services, among other community-based support strategies in Lesotho, South Africa, Namibia and Botswana [237]. Several of these interventions have simultaneously incorporated multiple strategies, such as the successful use of a multicomponent package among adult patients in Uganda [220]. Adherence patterns improved with a combination of approaches including counselling, group education, information leaflets, tracing of late attendees and the use of diaries to monitor adherence [220].

#### Interventions using economic incentives

The use of economic incentives has been evaluated among HIV-infected adults and caregivers of infected children in African settings. The introduction of food rations in Zambia [238] was associated with a demonstrable increase in ART adherence among adult patients in eight outpatient clinics. A recent initiative by Ssewamala *et al*. [239] in 32 clinics across Uganda is designed to enhance economic empowerment among youth with HIV, with the aim of improving ART adherence. This intervention, called “SUUBI+Adherence,” focuses on improving adherence self-efficacy in adolescents with HIV both in and out of school, through developing economic empowerment and financial management skills, as well as improving mental health among these youth [239]. Among other community support services, nutritional and cash support provided to patients attending outpatient clinics in Lesotho, South Africa, Namibia and Botswana resulted in improved clinical outcomes, including adherence, after 18 months [237].

### Guidelines relevant for adolescent adherence to HIV care

Effective interventions for adolescents may be different from those effective in adult populations. For example, based on findings from 325 studies conducted in both well-resourced and low-resource settings, the International Association of Physicians in AIDS Care (IAPAC) [239] recommends interventions offering therapeutic support using problem-solving techniques and addressing psychosocial contexts, for adolescent and youth populations. In addition, directly observed administration of ART (DAART) is recognized as potentially useful because it requires that treatment is not left to youth in isolation but involves the participation of caregivers. Although DAART is not recommended for adult populations in routine clinical care settings, there is evidence for its efficacy among paediatric and adolescent patients, and as such is recommended for this younger population, with other supportive interventions as adolescence progresses [240].

The US Department of Health and Human Services provides guidelines on ART use across age groups. Among measures for maximizing adherence in adolescents, it strongly recommends discussions of adherence-improvement strategies with the adolescent before initiating treatment, and at each treatment visit [241]. Also recommended is the use of adherence monitoring measures, and the maintenance of a non-judgmental, supportive provider–patient relationship [241]. In recognition of the challenges of pill burden, a once-daily ART regimen is also recommended, where feasible [241].

Interventions for adolescents with HIV may be successful if targeted both at them and their caregivers. Based on evaluations of a youth's competencies and challenges, caregiver competencies, and dynamics of the youth-caregiver relationship, strategies to improve youth self-management of adherence should be combined with arrangements to maintain caregiver involvement [242]. These become crucial as children grow into adolescence, particularly in the context of evolving peer relationships and the realities of stigma and discrimination. Challenges in this population may differ from those of younger children, and there is a need to design targeted interventions which are both efficacious and relevant to them.

## Conclusions

Children and adolescents living with HIV are a growing population in sub-Saharan Africa. During adolescence, accumulated barriers against the high levels of ART adherence necessary to maintain viral suppression and preserve health often threaten their physical and psychological wellbeing. Although much research has been conducted to identify such barriers and to target effective interventions to improve adherence, most studies have focused on adult patients.

Research findings suggest that care providers and caregivers may play a significant role in preventing adherence problems by addressing crucial issues early. Ensuring early commencement of the disclosure process, and monitoring psychosocial risks such as learning problems, social difficulties, psychological disorders and stressful experiences, particularly following the loss of loved ones, may help to provide support before the onset of adolescence, when youths are faced with additional developmental pressures. The partial transfer of treatment responsibility to the adolescent at this stage may result in better adherence behaviour if such support has been provided.

In sub-Saharan Africa, opportunities for communication and linkage with same-age peers living with HIV may also reduce isolation and provide valuable support in stressful circumstances, helping adolescents to achieve and maintain desirable adherence habits. As observed in well-resourced environments, adherence support by caregivers is likely essential until youth competency and responsibility are assured. Although non-age-specific strategies such as psychosocial interventions, economic incentives, DAART and other therapeutic support may be effective, there is need for further research to better understand the experiences of adolescents living with HIV/AIDS in sub-Saharan Africa, and inform the development of well-tailored culturally appropriate interventions to optimize ART adherence.

## References

[1] UNAIDS (2014). The gap report.

[2] World Health Organization (2013). Adolescent health.

[3] UNICEF (2011). The state of the world's children 2011: adolescence – an age of opportunity.

[4] Marcell AV, Kliegman RM, Behrman RE, Jenson HB, Stanton BMD, Zitelli BJ, Davies HW (2007). Adolescence. Nelson textbook of pediatrics.

[5] Gardner M, Steinberg L (2005). Peer influence on risk taking, risk preference, and risky decision making in adolescence and adulthood: an experimental study. Dev Psychol.

[6] Chein J, Albert D, O'Brien L, Uckert K, Steinberg L (2011). Peers increase adolescent risk taking by enhancing activity in the brain's reward circuitry. Dev Sci.

[7] UNFPA (2014). Adolescents and youth: towards realizing the full potential of adolescents and youth [Internet].

[8] Blum RW, Nelson-Mmari K (2004). The health of young people in a global context. J Adolesc Health.

[9] Romer D (2010). Adolescent risk taking, impulsivity, and brain development: implications for prevention. Dev Psychobiol.

[10] World Health Organization (2012). Adolescent mental health: mapping actions of nongovernmental organizations and other international development organizations [Internet].

[11] World Health Organization Dept of Child and Adolescent Health and Development (2003). Adolescent friendly health services: an agenda for change [Internet].

[12] Idele P, Gillespie A, Porth T, Suzuki C, Mahy M, Kasedde S (2014). Epidemiology of HIV and AIDS among adolescents: current status, inequities, and data gaps. J Acquir Immune Defic Syndr.

[13] Parienti J-J (2014). The case of adherence in the youth: rebel without a cause?. AIDS.

[14] Sawyer S, Drew S, Duncan R (2007). Adolescents with chronic disease – the double whammy. Aust Fam Physician.

[15] Bregnballe V, Schiøtz PO, Lomborg K (2011). Parenting adolescents with cystic fibrosis: the adolescents’ and young adults’ perspectives. Patient Prefer Adherence.

[16] World Health Organization (2007). The adolescent with a chronic condition.

[17] Patel V, Flisher AJ, Hetrick S, McGorry P (2007). Mental health of young people: a global public-health challenge. Lancet.

[18] UNAIDS (2013). AIDS by the numbers [Internet].

[19] UNICEF (2013). Towards an AIDS-free generation – children and AIDS: sixth stocktaking report, 2013.

[20] UNICEF (2014). All in towards ending the AIDS epidemic among adolescents [Internet].

[21] UNICEF (2010). Opportunity in crisis: preventing HIV from early adolescence to young adulthood.

[22] United Nations (2012). Regional overview: youth in Africa [Internet].

[23] UNICEF (2012). Progress for children: a report card on adolescents (No. 10).

[24] Zou J, Yamanaka Y, John M, Watt M, Ostermann J, Thielman N (2009). Religion and HIV in Tanzania: influence of religious beliefs on HIV stigma, disclosure, and treatment attitudes. BMC Public Health.

[25] Balogun AS (2010). Islamic perspectives on HIV/AIDS and antiretroviral treatment: the case of Nigeria. Afr J AIDS Res.

[26] Amuri M, Mitchell S, Cockcroft A, Andersson N (2011). Socio-economic status and HIV/AIDS stigma in Tanzania. AIDS Care.

[27] O'Brien S, Broom A (2013). Gender, culture and changing attitudes: experiences of HIV in Zimbabwe. Cult Health Sex.

[28] Roura M, Wringe A, Busza J, Nhandi B, Mbata D, Zaba B (2009). “Just like fever”: a qualitative study on the impact of antiretroviral provision on the normalisation of HIV in rural Tanzania and its implications for prevention. BMC Int Health Hum Rights.

[29] Rankin WW, Brennan S, Schell E, Laviwa J, Rankin SH (2005). The stigma of being HIV-positive in Africa. PLoS Med.

[30] Duff P, Kipp W, Wild TC, Rubaale T, Okech-Ojony J (2010). Barriers to accessing highly active antiretroviral therapy by HIV-positive women attending an antenatal clinic in a regional hospital in western Uganda. J Int AIDS Soc.

[31] Theilgaard ZP, Katzenstein TL, Chiduo MG, Pahl C, Bygbjerg IC, Gerstoft J (2011). Addressing the fear and consequences of stigmatization – a necessary step towards making HAART accessible to women in Tanzania: a qualitative study. AIDS Res Ther.

[32] Akullian A, Kohler P, Kinuthia J, Laserson K, Mills LA, Okanda J (2014). Geographic distribution of HIV stigma among women of childbearing age in rural Kenya. AIDS Lond Engl.

[33] Stangl AL, Lloyd JK, Brady LM, Holland CE, Baral S (2013). A systematic review of interventions to reduce HIV-related stigma and discrimination from 2002 to 2013: how far have we come?. J Int AIDS Soc.

[34] Kandwal R, Bahl T (2011). Link to slower access to care: what is the stigma?: an Indian perspective. Curr HIV/AIDS Rep.

[35] Williams MV, Palar K, Derose KP (2011). Congregation-based programs to address HIV/AIDS: elements of successful implementation. J Urban Health Bull N Y Acad Med.

[36] Wolf HT, Halpern-Felsher BL, Bukusi EA, Agot KE, Cohen CR, Auerswald CL (2014). “It is all about the fear of being discriminated [against] … the person suffering from HIV will not be accepted”: a qualitative study exploring the reasons for loss to follow-up among HIV-positive youth in Kisumu, Kenya. BMC Public Health.

[37] Madiba S, Mokgatle M (2015). “Students want HIV testing in schools” a formative evaluation of the acceptability of HIV testing and counselling at schools in Gauteng and North West provinces in South Africa. BMC Public Health.

[38] Denison JA, Banda H, Dennis AC, Packer C, Nyambe N, Stalter RM (2015). “The sky is the limit”: adhering to antiretroviral therapy and HIV self-management from the perspectives of adolescents living with HIV and their adult caregivers. J Int AIDS Soc.

[39] Birungi H, Obare F, van der Kwaak A, Namwebya JH (2011). Maternal health care utilization among HIV-positive female adolescents in Kenya. Int Perspect Sex Reprod Health.

[40] Centers for Disease Control and Prevention (2014). HIV among youth [Internet].

[41] UNICEF (2012). Preventing HIV infection among adolescents and young people [Internet].

[42] Muula AS (2008). HIV infection and AIDS among young women in South Africa. Croat Med J.

[43] UNICEF (2009). Eatern & Southern Africa HIV & AIDS – preventing HIV infection among adolescents and young people.

[44] Bankole A, Singh S, Woog V, Wulf D (2004). Risk and protection: youth and HIV/AIDS in sub-Saharan Africa [Internet].

[45] Muula AS, Ngulube TJ, Siziya S, Makupe CM, Umar E, Prozesky HW (2007). Gender distribution of adult patients on highly active antiretroviral therapy (HAART) in Southern Africa: a systematic review. BMC Public Health.

[46] UNAIDS (2014). Access to antiretroviral therapy in Africa: status report on progress towards the 2015 targets [Internet].

[47] UNAIDS (2014). Global AIDS response progress reporting.

[48] World Health Organization (2007). Towards universal access. Scaling up priority HIV/AIDS interventions in the health sector.

[49] UNAIDS (2013). Global report 2013 – UNAIDS global report 2013 [Internet].

[50] Hazra R, Siberry GK, Mofenson LM (2010). Growing up with HIV: children, adolescents, and young adults with perinatally acquired HIV infection. Annu Rev Med.

[51] Anaky M-F, Duvignac J, Wemin L, Kouakoussui A, Karcher S, Toure S (2010). Scaling up antiretroviral therapy for HIV-infected children in Cote d'Ivoire: determinants of survival and loss to programme. Bull World Health Organ.

[52] Davies M-A, Keiser O, Technau K, Eley B, Rabie H, van Cutsem G (2009). Outcomes of the South African National Antiretroviral Treatment Programme for children: the IeDEA Southern Africa collaboration. South Afr Med J.

[53] Van Dijk JH, Sutcliffe CG, Munsanje B, Sinywimaanzi P, Hamangaba F, Thuma PE (2011). HIV-infected children in rural Zambia achieve good immunologic and virologic outcomes two years after initiating antiretroviral therapy. PLoS One.

[54] Buchacz K, Rogol AD, Lindsey JC, Wilson CM, Hughes MD, Seage GR (2003). Delayed onset of pubertal development in children and adolescents with perinatally acquired HIV infection. J Acquir Immune Defic Syndr.

[55] Wood SM, Shah SS, Steenhoff AP, Rutstein RM (2009). The impact of AIDS diagnoses on long-term neurocognitive and psychiatric outcomes of surviving adolescents with perinatally acquired HIV. AIDS Lond Engl.

[56] Andiman WA, Chernoff MC, Mitchell C, Purswani M, Oleske J, Williams PL (2009). Incidence of persistent renal dysfunction in human immunodeficiency virus-infected children: associations with the use of antiretrovirals, and other nephrotoxic medications and risk factors. Pediatr Infect Dis J.

[57] Patel K, Mittleman M, Colan S, Oleske J, Patel K, Van Dyke R (2010). Predictors of cardiac dysfunction among children and adolescents perinatally-infected with HIV-1.

[58] Fakoya A, Lamba H, Mackie N (2008). British HIV Association, BASHH and FSRH guidelines for the management of the sexual and reproductive health of people living with HIV infection 2008. HIV Med.

[59] Cowan F, Pettifor A (2009). HIV in adolescents in sub-Saharan Africa. Curr Opin HIV AIDS.

[60] Kasedde S, Luo C, McClure C, Chandan U (2013). Reducing HIV and AIDS in adolescents: opportunities and challenges. Curr HIV/AIDS Rep.

[61] Strode A, Grant K (2011). Children and HIV: using an evidence-based approach to identifying legal strategies that protect and promote the right of children infected and affected by HIV and AIDS. Working paper prepared for the third meeting of the technical advisory group of the global commission on HIV and the law.

[62] World Health Organization (2013). HIV and adolescents: HIV testing and counselling, treatment and care for adolescents living with HIV: summary of key features and recommendations [Internet].

[63] World Health Organization (2014). Adolescent health epidemiology.

[64] World Health Organization (2014). WHO calls for stronger focus on adolescent health.

[65] Nichols SL, Bethel J, Garvie PA, Patton DE, Thornton S, Kapogiannis BG (2013). Neurocognitive functioning in antiretroviral therapy-naïve youth with behaviorally acquired human immunodeficiency virus. J Adolesc Health Off Publ Soc Adolesc Med.

[66] Smith R, Chernoff M, Williams PL (2012). Impact of human immunodeficiency virus severity on cognitive and adaptive functioning during childhood and adolescence. Pediatr Infect Dis J.

[67] Smith R, Wilkins M (2015). Perinatally acquired HIV infection: long-term neuropsychological consequences and challenges ahead. Child Neuropsychol.

[68] Mellins CA, Malee KM (2013). Understanding the mental health of youth living with perinatal HIV infection: lessons learned and current challenges. J Int AIDS Soc.

[69] Holguin A, Banda M, Willen EJ, Malama C, Chiyenu KO, Mudenda VC (2011). HIV-1 effects on neuropsychological performance in a resource-limited country, Zambia. AIDS Behav.

[70] Joska JA, Westgarth-Taylor J, Hoare J, Thomas KGF, Paul R, Myer L (2012). Neuropsychological outcomes in adults commencing highly active anti-retroviral treatment in South Africa: a prospective study. BMC Infect Dis.

[71] Nakasujja N, Skolasky RL, Musisi S, Allebeck P, Robertson K, Ronald A (2010). Depression symptoms and cognitive function among individuals with advanced HIV infection initiating HAART in Uganda. BMC Psychiatry.

[72] Patel VN, Mungwira RG, Tarumbiswa TF, Heikinheimo T, van Oosterhout JJ (2010). High prevalence of suspected HIV-associated dementia in adult Malawian HIV patients. Int J STD AIDS.

[73] Salawu FK, Bwala SA, Wakil MA, Bani B, Bukbuk DN, Kida I (2008). Cognitive function in HIV-seropositive Nigerians without AIDS. J Neurol Sci.

[74] Njamnshi AK, Djientcheu Vde P, Fonsah JY, Yepnjio FN, Njamnshi DM, Muna WE (2008). The International HIV Dementia Scale is a useful screening tool for HIV-associated dementia/cognitive impairment in HIV-infected adults in Yaoundé-Cameroon. J Acquir Immune Defic Syndr.

[75] Boivin MJ, Green SD, Davies AG, Giordani B, Mokili JK, Cutting WA (1995). A preliminary evaluation of the cognitive and motor effects of pediatric HIV infection in Zairian children. Health Psychol.

[76] Abubakar A, Van Baar A, Van de Vijver FJ, Holding P, Newton CR (2008). Paediatric HIV and neurodevelopment in sub-Saharan Africa: a systematic review. Trop Med Int Health.

[77] Bagenda D, Nassali A, Kalyesubula I, Sherman B, Drotar D, Boivin MJ (2006). Health, neurologic, and cognitive status of HIV-infected, long-surviving, and antiretroviral-naive Ugandan children. Pediatrics.

[78] Msellati P, Lepage P, Hitimana DG, Van Goethem C, Van de Perre P, Dabis F (1993). Neurodevelopmental testing of children born to human immunodeficiency virus type 1 seropositive and seronegative mothers: a prospective cohort study in Kigali, Rwanda. Pediatrics.

[79] World Health Organization (2003). Adherence to long-term therapies – evidence for action [Internet].

[80] Ajose O, Mookerjee S, Mills EJ, Boulle A, Ford N (2012). Treatment outcomes of patients on second-line antiretroviral therapy in resource-limited settings: a systematic review and meta-analysis. AIDS.

[81] Chesney MA (2006). The elusive gold standard. Future perspectives for HIV adherence assessment and intervention. J Acquir Immune Defic Syndr.

[82] Parienti J-J, Ragland K, Lucht F, de la Blanchardière A, Dargère S, Yazdanpanah Y (2010). Average adherence to boosted protease inhibitor therapy, rather than the pattern of missed doses, as a predictor of HIV RNA replication. Clin Infect Dis.

[83] Paterson DL, Swindells S, Mohr J, Brester M, Vergis EN, Squier C (2000). Adherence to protease inhibitor therapy and outcomes in patients with HIV infection. Ann Intern Med.

[84] Nachega JB, Hislop M, Nguyen H, Dowdy DW, Chaisson RE, Regensberg L (2009). Antiretroviral therapy adherence, virologic and immunologic outcomes in adolescents compared with adults in Southern Africa. J Acquir Immune Defic Syndr.

[85] Murphy DA, Wilson CM, Durako SJ, Muenz LR, Belzer M, Adolescent Medicine HIV/AIDS Research Network (2001). Antiretroviral medication adherence among the REACH HIV-infected adolescent cohort in the USA. AIDS Care.

[86] Belzer ME, Fuchs DN, Luftman GS, Tucker DJ (1999). Antiretroviral adherence issues among HIV-positive adolescents and young adults. J Adolesc Health.

[87] Farmer KC (1999). Methods for measuring and monitoring medication regimen adherence in clinical trials and clinical practice. Clin Ther.

[88] Fairman K, Motherall B (2000). Evaluating medication adherence: which measure is right for your program?. J Manag Care Pharm.

[89] Wiens MO, MacLeod S, Musiime V, Ssenyonga M, Kizza R, Bakeera-Kitaka S (2012). Adherence to antiretroviral therapy in HIV-positive adolescents in Uganda assessed by multiple methods: a prospective cohort study. Paediatr Drugs.

[90] Langat NT, Odero W, Gatongi P (2012). Antiretroviral drug adherence by HIV infected children attending Kericho District Hospital, Kenya. East Afr J Public Health.

[91] Kim S-H, Gerver SM, Fidler S, Ward H (2014). Adherence to antiretroviral therapy in adolescents living with HIV: systematic review and meta-analysis. AIDS Lond Engl.

[92] Dube N, Summers R, Tint K-S, Mayayise G (2012). A pharmacovigilance study of adults on highly active antiretroviral therapy, South Africa: 2007–2011. Pan Afr Med J.

[93] Ryscavage PA, Anderson EJ, Sutton SH, Reddy S, Taiwo B (2011). Clinical outcomes of adolescents and young adults in adult HIV care. J Acquir Immune Defic Syndr.

[94] AIDSInfo (2014). Adherence to antiretroviral therapy ∣ adult and adolescent ARV guidelines.

[95] Rudy BJ, Murphy DA, Harris DR, Muenz L, Ellen J, Adolescent Trials Network for HIV/AIDS Interventions (2009). Patient-related risks for nonadherence to antiretroviral therapy among HIV-infected youth in the United States: a study of prevalence and interactions. AIDS Patient Care STDs.

[96] Agwu AL, Fairlie L (2013). Antiretroviral treatment, management challenges and outcomes in perinatally HIV-infected adolescents. J Int AIDS Soc.

[97] Evans D, Menezes C, Mahomed K, Macdonald P, Untiedt S, Levin L (2013). Treatment outcomes of HIV-infected adolescents attending public-sector HIV clinics across Gauteng and Mpumalanga, South Africa. AIDS Res Hum Retroviruses.

[98] Nglazi MD, Kranzer K, Holele P, Kaplan R, Mark D, Jaspan H (2012). Treatment outcomes in HIV-infected adolescents attending a community-based antiretroviral therapy clinic in South Africa. BMC Infect Dis.

[99] Shroufi A, Gunguwo H, Dixon M, Nyathi M, Ndebele W, Saint-Sauveur J-F (2013). HIV-infected adolescents in southern Africa can achieve good treatment outcomes: results from a retrospective cohort study. AIDS Lond Engl.

[100] Bygrave H, Mtangirwa J, Ncube K, Ford N, Kranzer K, Munyaradzi D (2012). Antiretroviral therapy outcomes among adolescents and youth in rural Zimbabwe. PLoS One.

[101] Williams PL, Dyke RV, Eagle M, Smith D, Vincent C, Ciupak G (2008). Association of site-specific and participant-specific factors with retention of children in a long-term pediatric HIV cohort study. Am J Epidemiol.

[102] Dachew BA, Tesfahunegn TB, Birhanu AM (2014). Adherence to highly active antiretroviral therapy and associated factors among children at the University of Gondar Hospital and Gondar Poly Clinic, Northwest Ethiopia: a cross-sectional institutional based study. BMC Public Health.

[103] Chandwani S, Koenig LJ, Sill AM, Abramowitz S, Conner LC, D'Angelo L (2012). Predictors of antiretroviral medication adherence among a diverse cohort of adolescents with HIV. J Adolesc Health Off Publ Soc Adolesc Med.

[104] Mutwa PR, Van Nuil JI, Asiimwe-Kateera B (2013). Living situation affects adherence to combination antiretroviral therapy in HIV-infected adolescents in Rwanda: a qualitative study. PLoS One.

[105] Petersen I, Bhana A, Myeza N, Alicea S, John S, Holst H (2010). Psychosocial challenges and protective influences for socio-emotional coping of HIV+ adolescents in South Africa: a qualitative investigation. AIDS Care.

[106] de Carvalho FT, de Morais NA, Koller SH, Piccinini CA (2007). Protective factors and resilience in people living with HIV/AIDS. Cad Saúde Pública.

[107] Small L, Mercado M, Gopalan P, Pardo G, Ann Mellins C, McKay MM (2014). Enhancing the emotional wellbeing of perinatally HIV infected youth across global contexts. Glob Soc Welf Res Policy Pract.

[108] Sopena S, Evangeli M, Dodge J, Melvin D (2010). Coping and psychological adjustment in adolescents with vertically acquired HIV. AIDS Care.

[109] Harms S, Kizza R, Sebunnya J, Jack S (2009). Conceptions of mental health among Ugandan youth orphaned by AIDS. Afr J AIDS Res.

[110] Betancourt TS, Meyers-Ohki S, Stulac SN, Barrera AE, Mushashi C, Beardslee WR (2011). Nothing can defeat combined hands (Abashize hamwe ntakibananira): protective processes and resilience in Rwandan children and families affected by HIV/AIDS. Soc Sci Med.

[111] Mghamba FW, Minzi OMS, Massawe A, Sasi P (2013). Adherence to antiretroviral therapy among HIV infected children measured by caretaker report, medication return, and drug level in Dar Es Salaam, Tanzania. BMC Pediatr.

[112] Samet JH, Sullivan LM, Traphagen ET, Ickovics JR (2001). Measuring adherence among HIV-infected persons: is MEMS consummate technology?. AIDS Behav.

[113] Gross R, Tierney C, Andrade A (2009). Modified directly observed antiretroviral therapy compared with self-administered therapy in treatment-naive HIV-1-infected patients: a randomized trial. Arch Intern Med.

[114] Parsons GN, Siberry GK, Parsons JK, Christensen JR, Joyner ML, Lee SL (2006). Multidisciplinary, inpatient directly observed therapy for HIV-1-infected children and adolescents failing HAART: a retrospective study. AIDS Patient Care STDs.

[115] Glikman D, Walsh L, Valkenburg J, Mangat PD, Marcinak JF (2007). Hospital-based directly observed therapy for HIV-infected children and adolescents to assess adherence to antiretroviral medications. Pediatrics.

[116] Purdy JB, Freeman AF, Martin SC, Ryder C, Elliott-DeSorbo DK, Zeichner S (2008). Virologic response using directly observed therapy in adolescents with HIV: an adherence tool. J Assoc Nurses AIDS Care.

[117] Kaai S, Bullock S, Sarna A, Chersich M, Luchters S, Geibel S (2010). Perceived stigma among patients receiving antiretroviral treatment: a prospective randomised trial comparing an m-DOT strategy with standard-of-care in Kenya. SAHARA J.

[118] Munyao P, Luchters S, Chersich MF, Kaai S, Geibel S, Mandaliya KN (2010). Implementation of clinic-based modified-directly observed therapy (m-DOT) for ART; experiences in Mombasa, Kenya. AIDS Care.

[119] Vreeman RC, Nyandiko WM, Ayaya SO, Walumbe EG, Marrero DG, Inui TS (2010). The perceived impact of disclosure of pediatric HIV status on pediatric antiretroviral therapy adherence, child well-being, and social relationships in a resource-limited setting. AIDS Patient Care STDs.

[120] Nabukeera-Barungi N, Kalyesubula I, Kekitiinwa A, Byakika-Tusiime J, Musoke P (2007). Adherence to antiretroviral therapy in children attending Mulago Hospital, Kampala. Ann Trop Paediatr.

[121] Biressaw S, Abegaz WE, Abebe M, Taye WA, Belay M (2013). Adherence to antiretroviral therapy and associated factors among HIV infected children in Ethiopia: unannounced home-based pill count versus caregivers’ report. BMC Pediatr.

[122] Müller AD, Jaspan HB, Myer L, Hunter AL, Harling G, Bekker L-G (2011). Standard measures are inadequate to monitor pediatric adherence in a resource-limited setting. AIDS Behav.

[123] Haberer JE, Kiwanuka J, Nansera D, Wilson IB, Bangsberg DR (2010). Challenges in using mobile phones for collection of antiretroviral therapy adherence data in a resource-limited setting. AIDS Behav.

[124] Haberer JE, Kiwanuka J, Nansera D, Muzoora C, Hunt PW, So J (2013). Real-time adherence monitoring of antiretroviral therapy among HIV-infected adults and children in rural Uganda. AIDS.

[125] Muller AD, Bode S, Myer L, Roux P, von Steinbuchel N (2008). Electronic measurement of adherence to pediatric antiretroviral therapy in South Africa. Pediatr Infect Dis J.

[126] Haberer JE, Kiwanuka J, Nansera D, Ragland K, Mellins C, Bangsberg DR (2012). Multiple measures reveal antiretroviral adherence successes and challenges in HIV-infected Ugandan children. PLoS One.

[127] Kalichman SC, Amaral CM, Stearns H, White D, Flanagan J, Pope H (2007). Adherence to antiretroviral therapy assessed by unannounced pill counts conducted by telephone. J Gen Intern Med.

[128] Chesney MA (2000). Factors affecting adherence to antiretroviral therapy. Clin Infect Dis.

[129] Ndiaye M, Nyasulu P, Nguyen H, Lowenthal ED, Gross R, Mills EJ (2013). Risk factors for suboptimal antiretroviral therapy adherence in HIV-infected adolescents in Gaborone, Botswana: a pilot cross-sectional study. Patient Prefer Adherence.

[130] Bangsberg DR, Hecht FM, Charlebois ED, Chesney M, Moss A (2001). Comparing objective measures of adherence to HIV antiretroviral therapy: electronic medication monitors and unannounced pill counts. AIDS Behav.

[131] Hong SY, Jerger L, Jonas A (2013). Medication possession ratio associated with short-term virologic response in individuals initiating antiretroviral therapy in Namibia. PLoS One.

[132] Messou E, Chaix M-L, Gabillard D (2011). Association between medication possession ratio, virologic failure and drug resistance in HIV-1 infected adults on antiretroviral therapy in Côte d'Ivoire. J Acquir Immune Defic Syndr.

[133] Chi BH, Cantrell RA, Zulu I (2009). Adherence to first-line antiretroviral therapy affects non-virologic outcomes among patients on treatment for more than 12 months in Lusaka, Zambia. Int J Epidemiol.

[134] McMahon JH, Jordan MR, Kelley K, Bertagnolio S, Hong SY, Wanke CA (2011). Pharmacy adherence measures to assess adherence to antiretroviral therapy: review of the literature and implications for treatment monitoring. Clin Infect Dis.

[135] Jimmy-Gama D, Gibson S, McPake B, Maleta K (2011). Antiretroviral therapy (ART) rationing and access mechanisms and their impact on youth ART utilization in Malawi. Malawi Med J.

[136] Fetzer BC, Mupenda B, Lusiama J, Kitetele F, Golin C, Behets F (2011). Barriers to and facilitators of adherence to pediatric antiretroviral therapy in a sub-Saharan setting: insights from a qualitative study. AIDS Patient Care STDs.

[137] Biadgilign S, Deribew A, Amberbir A, Deribe K (2009). Barriers and facilitators to antiretroviral medication adherence among HIV-infected paediatric patients in Ethiopia: a qualitative study. SAHARA J.

[138] Arage G, Tessema G, Kassa H (2014). Adherence to antiretroviral therapy and its associated factors among children at South Wollo Zone Hospitals, Northeast Ethiopia: a cross-sectional study. BMC Public Health.

[139] Biadgilign S, Deribew A, Amberbir A, Deribe K (2008). Adherence to highly active antiretroviral therapy and its correlates among HIV infected pediatric patients in Ethiopia. BMC Pediatr.

[140] Rutengwe RM (2004). Identifying strategic interventions for improving household food and nutrition security in an urban informal settlement, South Africa. Asia Pac J Clin Nutr.

[141] Nyandiko WM, Ayaya S, Nabakwe E, Tenge C, Sidle JE, Yiannoutsos CT (2006). Outcomes of HIV-infected orphaned and non-orphaned children on antiretroviral therapy in western Kenya. J Acquir Immune Defic Syndr.

[142] Vreeman RC, Wiehe SE, Ayaya SO, Musick BS, Nyandiko WM (2008). Association of antiretroviral and clinic adherence with orphan status among HIV-infected children in Western Kenya. J Acquir Immune Defic Syndr.

[143] Bell S, Prata N, Lahiff M, Eskenazi B (2012). Civil unrest and birthweight: an exploratory analysis of the 2007/2008 Kenyan Crisis. Soc Sci Med.

[144] Mann M, Lurie MN, Kimaiyo S, Kantor R (2013). Effects of political conflict-induced treatment interruptions on HIV drug resistance. AIDS Rev.

[145] Zwi A, Ugalde A (1989). Towards an epidemiology of political violence in the third world. Soc Sci Med.

[146] Ityavyar DA, Ogba LO (1989). Violence, conflict and health in Africa. Soc Sci Med.

[147] Yach D (1988). The impact of political violence on health and health services in Cape Town, South Africa, 1986: methodological problems and preliminary results. Am J Public Health.

[148] Herman AA (1988). Political violence, health, and health services in South Africa. Am J Public Health.

[149] Pyne-Mercier LD, John-Stewart GC, Richardson BA, Kagondu NL, Thiga J, Noshy H (2011). The consequences of post-election violence on antiretroviral HIV therapy in Kenya. AIDS Care.

[150] Mann M, Diero L, Kemboi E (2013). Antiretroviral treatment interruptions induced by the Kenyan postelection crisis are associated with virological failure. J Acquir Immune Defic Syndr.

[151] Reid T, van Engelgem I, Telfer B, Manzi M (2008). Providing HIV care in the aftermath of Kenya's post-election violence Medecins Sans Frontieres’ lessons learned January – March 2008. Confl Health.

[152] Vreeman RC, Nyandiko WM, Ayaya SO, Walumbe EG, Marrero DG, Inui TS (2009). Factors sustaining pediatric adherence to antiretroviral therapy in Western Kenya. Qual Health Res.

[153] Bikaako-Kajura W, Luyirika E, Purcell DW, Downing J, Kaharuza F, Mermin J (2006). Disclosure of HIV status and adherence to daily drug regimens among HIV-infected children in Uganda. AIDS Behav.

[154] Peterson K, Togun T, Klis S, Menten J, Colebunders R (2012). Depression and posttraumatic stress disorder among HIV-infected Gambians on antiretroviral therapy. AIDS Patient Care STDs.

[155] Martin S, Elliott-DeSorbo DK, Wolters PL, Toledo-Tamula MA, Roby G, Zeichner S (2007). Patient, caregiver and regimen characteristics associated with adherence to highly active antiretroviral therapy among HIV-infected children and adolescents. Pediatr Infect Dis J.

[156] Murphy DA, Sarr M, Durako SJ, Moscicki A, Wilson CM, Muenz LR (2003). Barriers to HAART adherence among human immunodeficiency virus-infected adolescents. Arch Pediatr Adolesc Med.

[157] Hosek SG, Harper GW, Domanico R (2005). Predictors of medication adherence among HIV-infected youth. Psychol Health Med.

[158] MacDonell KE, Naar-King S, Murphy DA, Parsons JT, Huszti H (2011). Situational temptation for HIV medication adherence in high-risk youth. AIDS Patient Care STDs.

[159] MacDonell K, Naar-King S, Huszti H, Belzer M (2013). Barriers to medication adherence in behaviorally and perinatally infected youth living with HIV. AIDS Behav.

[160] Siu GE, Bakeera-Kitaka S, Kennedy CE, Dhabangi A, Kambugu A (2012). HIV serostatus disclosure and lived experiences of adolescents at the Transition Clinic of the Infectious Diseases Clinic in Kampala, Uganda: a qualitative study. AIDS Care.

[161] Vreeman RC, Gramelspacher AM, Gisore PO, Scanlon ML, Nyandiko WM (2013). Disclosure of HIV status to children in resource-limited settings: a systematic review. J Int AIDS Soc.

[162] Wiener L, Mellins CA, Marhefka S, Battles HB (2007). Disclosure of an HIV diagnosis to children: history, current research, and future directions. J Dev Behav Pediatr.

[163] Menon A, Glazebrook C, Campain N, Ngoma M (2007). Mental health and disclosure of HIV status in Zambian adolescents with HIV infection: implications for peer-support programs. J Acquir Immune Defic Syndr.

[164] Mburu G, Hodgson I, Kalibala S, Haamujompa C, Cataldo F, Lowenthal ED (2014). Adolescent HIV disclosure in Zambia: barriers, facilitators and outcomes. J Int AIDS Soc.

[165] Hejoaka F (2009). Care and secrecy: being a mother of children living with HIV in Burkina Faso. Soc Sci Med.

[166] Alubo O, Zwandor A, Jolayemi T, Omudu E (2002). Acceptance and stigmatization of PLWA in Nigeria. AIDS Care.

[167] Makoae LN, Portillo CJ, Uys LR (2009). The impact of taking or not taking ARVs on HIV stigma as reported by persons living with HIV infection in five African countries. AIDS Care.

[168] Elise A, France AM, Louise WM, Bata D, François R, Roger S, Philippe M (2005). Assessment of adherence to highly active antiretroviral therapy in a cohort of African HIV-infected children in Abidjan, Côte d'Ivoire. J Acquir Immune Defic Syndr.

[169] Iroha E, Esezobor CI, Ezeaka C, Temiye EO, Akinsulie A (2010). Adherence to antiretroviral therapy among HIV-infected children attending a donor-funded clinic at a tertiary hospital in Nigeria. Afr J AIDS Res.

[170] Mukhtar-Yola M, Adeleke S, Gwarzo D, Ladan ZF (2006). Preliminary investigation of adherence to antiretroviral therapy among children in Aminu Kano Teaching Hospital, Nigeria. Afr J AIDS Res.

[171] Polisset J, Ametonou F, Arrive E, Aho A, Perez F (2009). Correlates of adherence to antiretroviral therapy in HIV-infected children in Lomé, Togo, West Africa. AIDS Behav.

[172] Ugwu R, Eneh A (2013). Factors influencing adherence to paediatric antiretroviral therapy in Portharcourt, South- South Nigeria. Pan Afr Med J.

[173] Byakika-Tusiime J, Crane J, Oyugi JH, Ragland K, Kawuma A, Musoke P (2009). Longitudinal antiretroviral adherence in HIV+ Ugandan parents and their children initiating HAART in the MTCT-plus family treatment model: role of depression in declining adherence over time. AIDS Behav.

[174] Wamalwa DC, Farquhar C, Obimbo EM (2007). Early response to highly active antiretroviral therapy in HIV-1–infected Kenyan children. J Acquir Immune Defic Syndr.

[175] Reddi A, Leeper SC, Grobler AC (2007). Preliminary outcomes of a paediatric highly active antiretroviral therapy cohort from KwaZulu-Natal, South Africa. BMC Pediatr.

[176] Tsai AC, Bangsberg DR, Bwana M, Haberer JE, Frongillo EA, Muzoora C (2013). How does antiretroviral treatment attenuate the stigma of HIV? Evidence from a cohort study in rural Uganda. AIDS Behav.

[177] Gilbert L, Walker L (2009). “They (ARVs) are my life, without them I'm nothing” – experiences of patients attending a HIV/AIDS clinic in Johannesburg, South Africa. Health Place.

[178] Campbell C, Skovdal M, Madanhire C, Mugurungi O, Gregson S, Nyamukapa C (2011). “We, the AIDS people …”: how antiretroviral therapy enables Zimbabweans living with HIV/AIDS to cope with stigma. Am J Public Health.

[179] Zuch M, Lurie M (2012). “A virus and nothing else”: the effect of ART on HIV-related stigma in rural South Africa. AIDS Behav.

[180] Kaishusha Mupendwa BP, Kadima Ntokamunda JL (2009). Treatment adhesion and factors affecting it at the Kadutu Clinic (Democratic Republic of the Congo). Santé.

[181] Laughton B, Cornell M, Boivin M, Van Rie A (2013). Neurodevelopment in perinatally HIV-infected children: a concern for adolescence. J Int AIDS Soc.

[182] Dawes S, Suarez P, Casey CY, Cherner M, Marcotte TD, Letendre S (2008). Variable patterns of neuropsychological performance in HIV-1 infection. J Clin Exp Neuropsychol.

[183] Hoare J, Fouche J-P, Spottiswoode B (2012). A diffusion tensor imaging and neurocognitive study of HIV-positive children who are HAART-naïve “slow progressors”. J Neurovirol.

[184] Hoare J, Jacqueline H, Westgarth-Taylor J (2012). A diffusion tensor imaging and neuropsychological study of prospective memory impairment in South African HIV positive individuals. Metab Brain Dis.

[185] Abubakar A, Holding P, Van Baar A, Newton CR, Van de Vijver FJ, Espy KA (2013). The performance of children prenatally exposed to HIV on the A-Not-B task in Kilifi, Kenya: a preliminary study. Int J Environ Res Public Health.

[186] Melrose RJ, Tinaz S, Castelo JMB, Courtney MG, Stern CE (2008). Compromised fronto-striatal functioning in HIV: an fMRI investigation of semantic event sequencing. Behav Brain Res.

[187] Kandawasvika GQ, Ogundipe E, Gumbo FZ, Kurewa EN, Mapingure MP, Stray-Pedersen B (2011). Neurodevelopmental impairment among infants born to mothers infected with human immunodeficiency virus and uninfected mothers from three peri-urban primary care clinics in Harare, Zimbabwe. Dev Med Child Neurol.

[188] Nichols SL, Montepiedra G, Farley JJ, Sirois PA, Malee K, Kammerer B (2012). Cognitive, academic and behavioral correlates of medication adherence in children and adolescents with perinatally acquired HIV infection. J Dev Behav Pediatr.

[189] Rice ML, Buchanan AL, Siberry GK (2012). Language impairment in children perinatally infected with HIV compared to children who were HIV-exposed and uninfected. J Dev Behav Pediatr.

[190] Rice ML, Zeldow B, Siberry GK (2013). Evaluation of risk for late language emergence after *in utero* antiretroviral drug exposure in HIV-exposed uninfected infants. Pediatr Infect Dis J.

[191] Garvie PA, Zeldow B, Malee K, Nichols SL, Smith RA, Wilkins ML (2014). Discordance of cognitive and academic achievement outcomes in youth with perinatal HIV exposure. Pediatr Infect Dis J.

[192] Kandawasvika GQ, Kuona P, Chandiwana P, Masanganise M, Gumbo FZ, Mapingure MP (2015). The burden and predictors of cognitive impairment among 6- to 8-year-old children infected and uninfected with HIV from Harare, Zimbabwe: a cross-sectional study. Child Neuropsychol.

[193] Pugatch D, Bennett L, Patterson D (2002). HIV medication adherence in adolescents. J HIVAIDS Prev Educ Adolesc Child.

[194] Ford N, Lee J, Andrieux-Meyer I, Calmy A (2011). Safety, efficacy, and pharmacokinetics of rilpivirine: systematic review with an emphasis on resource-limited settings. HIVAIDS.

[195] Spreen WR, Margolis DA, Pottage JC (2013). Long-acting injectable antiretrovirals for HIV treatment and prevention. Curr Opin HIV AIDS.

[196] Spreen W, Williams P, Margolis D, Ford SL, Crauwels H, Lou Y (2014). Pharmacokinetics, safety, and tolerability with repeat doses of GSK1265744 and rilpivirine (TMC278) long-acting nanosuspensions in healthy adults. J Acquir Immune Defic Syndr.

[197] Cervia JS (2013). Easing the transition of HIV-infected adolescents to adult care. AIDS Patient Care STDs.

[198] Dowshen N, D'Angelo L (2011). Health care transition for youth living with HIV/AIDS. Pediatrics.

[199] Hussen SA, Chahroudi A, Boylan A, Camacho-Gonzalez AF, Hackett S, Chakraborty R (2015). Transition of youth living with HIV from pediatric to adult-oriented healthcare: a review of the literature. Future Virol.

[200] Wiener LS, Kohrt B-A, Battles HB, Pao M (2011). The HIV experience: youth identified barriers for transitioning from pediatric to adult care. J Pediatr Psychol.

[201] Malee K, Williams PL, Montepiedra G, Nichols S, Sirois PA, Storm D (2009). The role of cognitive functioning in medication adherence of children and adolescents with HIV infection. J Pediatr Psychol.

[202] Vijayan T, Benin AL, Wagner K, Romano S, Andiman WA (2009). We never thought this would happen: transitioning care of adolescents with perinatally acquired HIV infection from pediatrics to internal medicine. AIDS Care.

[203] Gilliam PP, Ellen JM, Leonard L, Kinsman S, Jevitt CM, Straub DM (2011). Transition of adolescents with HIV to adult care: characteristics and current practices of the adolescent trials network for HIV/AIDS interventions. J Assoc Nurses AIDS Care.

[204] Abaasa AM, Todd J, Ekoru K, Kalyango JN, Levin J, Odeke E (2008). Good adherence to HAART and improved survival in a community HIV/AIDS treatment and care programme: the experience of the AIDS Support Organization (TASO), Kampala, Uganda. BMC Health Serv Res.

[205] Mills EJ, Bakanda C, Birungi J, Chan K, Ford N, Cooper CL (2011). Life expectancy of persons receiving combination antiretroviral therapy in low-income countries: a cohort analysis from Uganda. Ann Intern Med.

[206] Lowenthal ED, Bakeera-Kitaka S, Marukutira T, Chapman J, Goldrath K, Ferrand RA (2014). Perinatally acquired HIV infection in adolescents from sub-Saharan Africa: a review of emerging challenges. Lancet Infect Dis.

[207] Attia S, Egger M, Müller M, Zwahlen M, Low N (2009). Sexual transmission of HIV according to viral load and antiretroviral therapy: systematic review and meta-analysis. AIDS Lond Engl.

[208] Bangsberg DR, Acosta EP, Gupta R, Guzman D, Riley ED, Harrigan PR (2006). Adherence-resistance relationships for protease and non-nucleoside reverse transcriptase inhibitors explained by virological fitness. AIDS Lond Engl.

[209] Wright MJ, Woo E, Foley J (2011). Antiretroviral adherence and the nature of HIV-associated verbal memory impairment. J Neuropsychiatry Clin Neurosci.

[210] Hinkin CH, Hardy DJ, Mason KI, Castellon SA, Durvasula RS, Lam MN (2004). Medication adherence in HIV-infected adults: effect of patient age, cognitive status, and substance abuse. AIDS.

[211] Simoni JM, Frick PA, Pantalone DW, Turner BJ (2003). Antiretroviral adherence interventions: a review of current literature and ongoing studies. Top HIV Med.

[212] Scanlon ML, Vreeman RC (2013). Current strategies for improving access and adherence to antiretroviral therapies in resource-limited settings. HIVAIDS.

[213] Simoni JM, Pearson CR, Pantalone DW, Marks G, Crepaz N (2006). Efficacy of interventions in improving highly active antiretroviral therapy adherence and HIV-1 RNA viral load. J Acquir Immune Defic Syndr.

[214] Rueda S, Park-Wyllie LY, Bayoumi AM, Tynan AM, Antoniou TA, Rourke SB (2006). Patient support and education for promoting adherence to highly active antiretroviral therapy for HIV/AIDS. Cochrane Database Syst Rev.

[215] Amico KR, Harman JJ, Johnson BT (2006). Efficacy of antiretroviral therapy adherence interventions: a research synthesis of trials, 1996 to 2004. J Acquir Immune Defic Syndr.

[216] Van Winghem J, Telfer B, Reid T, Ouko J, Mutunga A, Jama Z (2008). Implementation of a comprehensive program including psycho-social and treatment literacy activities to improve adherence to HIV care and treatment for a pediatric population in Kenya. BMC Pediatr.

[217] Kenu E, Obo-Akwa A, Nuamah GB, Brefo A, Sam M, Lartey M (2014). Knowledge and disclosure of HIV status among adolescents and young adults attending an adolescent HIV clinic in Accra, Ghana. BMC Res Notes.

[218] Workneh G, Scherzer L, Kirk B, Draper HR, Anabwani G, Wanless RS (2013). Evaluation of the effectiveness of an outreach clinical mentoring programme in support of paediatric HIV care scale-up in Botswana. AIDS Care.

[219] Dowse R, Barford K, Browne SH (2014). Simple, illustrated medicines information improves ARV knowledge and patient self-efficacy in limited literacy South African HIV patients. AIDS Care.

[220] Kunutsor S, Walley J, Muchuro S, Katabira E, Balidawa H, Namagala E (2012). Improving adherence to antiretroviral therapy in sub-Saharan African HIV-positive populations: an enhanced adherence package. AIDS Care.

[221] Navarra A-M, Neu N, Toussi S, Nelson J, Larson EL (2014). Health literacy and adherence to antiretroviral therapy among HIV-infected youth. J Assoc Nurses AIDS Care.

[222] Garvie PA, Lensing S, Rai SN (2007). Efficacy of a pill-swallowing training intervention to improve antiretroviral medication adherence in pediatric patients with HIV/AIDS. Pediatrics.

[223] MacPherson P, Munthali C, Ferguson J, Armstrong A, Kranzer K, Ferrand RA (2015). Service delivery interventions to improve adolescents’ linkage, retention and adherence to antiretroviral therapy and HIV care. Trop Med Int Health.

[224] Greifinger R, Dick B (2011). Provision of psychosocial support for young people living with HIV: voices from the field. SAHARA J.

[225] Crozatti MTL, França-Junior I, Rodrigues R, Carneiro Ferrão Mdo S, Brigido LF, Della Negra M (2013). Antiretroviral treatment adherence in childhood and adolescence: multidisciplinary team as an associated factor in Brazil. AIDS Care.

[226] Garvie PA, Lawford J, Flynn PM, Gaur AH, Belzer M, McSherry GD (2009). Development of a directly observed therapy adherence intervention for adolescents with human immunodeficiency virus-1: application of focus group methodology to inform design, feasibility, and acceptability. J Adolesc Health Off Publ Soc Adolesc Med.

[227] Lester RT, Ritvo P, Mills EJ (2010). Effects of a mobile phone short message service on antiretroviral treatment adherence in Kenya (WelTel Kenya1): a randomised trial. Lancet.

[228] Pop-Eleches C, Thirumurthy H, Habyarimana JP (2011). Mobile phone technologies improve adherence to antiretroviral treatment in a resource-limited setting: a randomized controlled trial of text message reminders. AIDS Lond Engl.

[229] Sarna A, Luchters S, Geibel S, Chersich MF, Munyao P, Kaai S (2008). Short- and long-term efficacy of modified directly observed antiretroviral treatment in Mombasa, Kenya: a randomized trial. J Acquir Immune Defic Syndr.

[230] Pearson CR, Micek MA, Simoni JM, Hoff PD, Matediana E, Martin DP (2007). Randomized control trial of peer-delivered, modified directly observed therapy for HAART in Mozambique. J Acquir Immune Defic Syndr.

[231] Nachega JB, Chaisson RE, Goliath R, Efron A, Chaudhary MA, Ram M (2010). Randomized controlled trial of trained patient-nominated treatment supporters providing partial directly observed antiretroviral therapy. AIDS.

[232] Idoko JA, Agbaji O, Agaba P (2007). Direct observation therapy-highly active antiretroviral therapy in a resource-limited setting: the use of community treatment support can be effective. Int J STD AIDS.

[233] Taiwo BOM, Idoko JAM, Welty LJ, Otoh I, Job GR, Iyaji PG (2010). Assessing the viorologic and adherence benefits of patient-selected HIV treatment partners in a resource-limited setting. J Acquir Immune Defic Syndr.

[234] Bärnighausen T, Chaiyachati K, Chimbindi N, Peoples A, Haberer J, Newell M-L (2011). Interventions to increase antiretroviral adherence in sub-Saharan Africa: a systematic review of evaluation studies. Lancet Infect Dis.

[235] Chang LW, Kagaayi J, Nakigozi G, Packer AH, Serwadda D, Quinn TC (2008). Responding to the human resource crisis: peer health workers, mobile phones, and HIV care in Rakai, Uganda. AIDS Patient Care STDs.

[236] Stubbs BA, Micek MA, Pfeiffer JT, Montoya P, Gloyd S (2009). Treatment partners and adherence to HAART in Central Mozambique. AIDS Care.

[237] Kabore I, Bloem J, Etheredge G (2010). The effect of community-based support services on clinical efficacy and health-related quality of life in HIV/AIDS patients in resource-limited settings in sub-Saharan Africa. AIDS Patient Care STDs.

[238] Cantrell RA, Sinkala M, Megazinni K (2008). A pilot study of food supplementation to improve adherence to antiretroviral therapy among food-insecure adults in Lusaka, Zambia. J Acquir Immune Defic Syndr.

[239] Ssewamala FM, Nabunya P, Ilic V, Mukasa MN, Ddamulira C (2015). Relationship between family economic resources, psychosocial well-being, and educational preferences of AIDS-orphaned children in southern uganda: baseline findings. Glob Soc Welf Res Policy Pract.

[240] Thompson MA, Mugavero MJ, Amico KR (2012). Guidelines for improving entry into and retention in care and antiretroviral adherence for persons with HIV: evidence-based recommendations from an International Association of Physicians in AIDS care panel. Ann Intern Med.

[241] U.S. Department of Health and Human Services (2014). Guidelines for the use of antiretroviral agents in pediatric HIV infection.

[242] Elkington KS, Bauermeister JA, Robbins RN, Gromadzka O, Abrams EJ, Wiznia A (2012). Individual and contextual factors of sexual risk behavior in youth perinatally infected with HIV. AIDS Patient Care STDs.

[243] Musiime V, Kizito H, Ssali F, Namusoke A, Mugisha M, Kityo C (2007). An adolescent peer support group improves adherence to antiretroviral therapy and reduces self-stigma among HIV-infected adolescents at joint clinical research centre (JCRC), Kampala.

[244] Bhana A, Mellins CA, Petersen I (2014). The VUKA family program: piloting a family-based psychosocial intervention to promote health and mental health among HIV infected early adolescents in South Africa. AIDS Care.

[245] Fatti G, Shaikh N, Eley B, Grimwood A (2014). Improved virological suppression in children on antiretroviral treatment receiving community-based adherence support: a multicentre cohort study from South Africa. AIDS Care.

[246] Mavhu W, Berwick J, Chirawu P (2013). Enhancing psychosocial support for HIV positive adolescents in Harare, Zimbabwe. PLoS One.

